# Why Do Tropical Mountains Support Exceptionally High Biodiversity? The Eastern Arc Mountains and the Drivers of *Saintpaulia* Diversity

**DOI:** 10.1371/journal.pone.0048908

**Published:** 2012-11-19

**Authors:** Dimitar Dimitrov, David Nogués-Bravo, Nikolaj Scharff

**Affiliations:** 1 Center for Macroecology, Evolution and Climate, Natural History Museum of Denmark, Zoological Museum, University of Copenhagen, Copenhagen, Denmark; 2 Center for Macroecology, Evolution and Climate, University of Copenhagen, Copenhagen, Denmark; CNR, Italy

## Abstract

We combine information about the evolutionary history and distributional patterns of the genus *Saintpaulia* H. Wendl. (Gesneriaceae; ‘African violets’) to elucidate the factors and processes behind the accumulation of species in tropical montane areas of high biodiversity concentration. We find that high levels of biodiversity in the Eastern Arc Mountains are the result of pre-Quaternary speciation processes and environmental stability. Our results support the hypothesis that climatically stable mountaintops may have acted as climatic refugia for lowland lineages during the Pleistocene by preventing extinctions. In addition, we found evidence for the existence of lowland micro-refugia during the Pleistocene, which may explain the high species diversity of East African coastal forests. We discuss the conservation implications of the results in the context of future climate change.

## Introduction

The processes that have led to the accumulation of species in hotspots of biological diversity continue to be elusive [1], [2]. For tropical mountains, two different scenarios have been proposed: Pleistocene glacial refugia [3]–[6] and the long-term stability [7]. The refugia model suggests that differences in species diversity between refugia are the result of allopatric speciation and a reduced level of extinctions due to reduced climatic fluctuations within refugia. New species resulting from this process should also have allopatric distributions, which in the context of tropical mountains implies that different species should be present on different mountain blocks. Stability, in contrast, reduces extinction rates within an area and permits survival of relictual lineages; thus species richness is not a result of extinctions outside of stable areas. Meanwhile, stability does not preclude sympatric speciation of radiating groups within these areas. Under the stability scenario young species are expected to be distributed around the periphery of stable areas where contractions, extensions and/or temporary fragmentation of these areas has increased heterogeneity. We explore these hypotheses using the biodiversity hotspot in the Eastern Arc Mountains of Tanzania and Kenya and focus on the genus *Saintpaulia* H. Wendl. (Gesneriaceae; ‘African violets’) as a study system.

The discovery of extensive numbers of endemics with regional distribution seems to support the long-term stability model. Furthermore, data from sediment cores from the Uluguru Mountains provides evidence that highland forest composition has remained stable for at least the last 48000 years [8], which might suggest similarly stable conditions across the earlier glacial-interglacial cycles during the Pleistocene. These results are in concordance with previous findings from sedimentary cores from the larger East African region (see also [4], [9]). Emerging evidence that local climatic conditions in the eastern Arc have been stable during periods of past global climatic changes provides a possible explanation for the extremely rich biota and numerous endemic species found in the region (e.g., [7], [10]). Much of the tree species diversity in East African forests has been linked to vicariance due to the pan-African tropical forest fragmentation initiated during the Oligocene-Early Miocene [10]. A similar pattern has been reported for amphibians [11] and in both cases lack of diversification during Pleistocene climate oscillations has been attributed to the stable conditions on mountain tops. Likewise, much of the bird diversity in the Eastern Arc stems from speciation events predating the Pleistocene and is consistent with repeated vicariance and dispersal events in expanding and contracting forests, which supports a model of long-term stability in the face of climatic fluctuations [12]. Recently Measey and Tolley [13] have shown that effects of forest fragmentation and local expansions and contractions can be detected in the Taita Hills leaf chameleon’s biogeographical history. In some cases differentiation among populations was attributed to fluctuations in forest extent during the Pleistocene, and – in concordance with the stability hypothesis – new lineages formed on the periphery of stable areas.

**Table 1:** Accession numbers of *Saintpaulia* sequences used in the analyses and specimen vouchers accession numbers and depositories.

GenBank accession	Old name	Name sensu Darbyshire (2006)	Voucher
AF307030	*Saintpaulia goetzeana*	*Saintpaulia goetzeana* 1	1997-1201 E
AF108735	*Saintpaulia goetzeana*	*Saintpaulia goetzeana* 2	Kilangala.1 EA_NMK
AF108734	*Saintpaulia goetzeana*	*Saintpaulia goetzeana* 3	Lupanga.1 EA_NMK
AF108731	*Saintpaulia* cf. *nitida*	*Saintpaulia ionantha* cf. subsp. *nitida* 3 *	Kwamtili.4 EA_NMK
AF307031	*Saintpaulia grandifolia*	*Saintpaulia ionantha* subsp. *grandifolia* 4	1985-0678 E
AF307021	*Saintpaulia grandifolia*	*Saintpaulia ionantha* subsp. *grandifolia* 5	Nkoloi Stream.3 EA_NMK
AF324926	*Saintpaulia difficilis*	*Saintpaulia ionantha* subsp. *grotei* 6	1972-1415 E
AF307039	*Saintpaulia magungensis* var. *occidentalis*	*Saintpaulia ionantha* subsp. *grotei* 7	1985-0680 E
AF307038	*Saintpaulia magungensis* var. *minima*	*Saintpaulia ionantha* subsp. *grotei* 8	1959-4352 E
AF307037	*Saintpaulia magungensis*	*Saintpaulia ionantha* subsp. *grotei* 9	1992-3187 E
AF307032	*Saintpaulia grotei*	*Saintpaulia ionantha* subsp. *grotei* 10	1987-2171 E
AF307028	*Saintpaulia difficilis*	*Saintpaulia ionantha* subsp. *grotei* 11	1987-2176 E
AF307027	*Saintpaulia confusa*	*Saintpaulia ionantha* subsp. *grotei* 12	JS13 E
AF307019	*Saintpaulia difficilis*	*Saintpaulia ionantha* subsp. *grotei* 13	Kiganga1 EA_NMK
AF108738	*Saintpaulia magungensis* var. *minima*	*Saintpaulia ionantha* subsp. *grotei* 14	1963-42311 K
AF108737	*Saintpaulia magungensis*	*Saintpaulia ionantha* subsp. *grotei* 15	Magunga.4 EA_NMK
AF108729	*Saintpaulia grotei*	*Saintpaulia ionantha* subsp. *grotei* 16	1995-511 K
AF108728	*Saintpaulia confusa*	*Saintpaulia ionantha* subsp. *grotei* 17	1974-2873 K
AF307050	*Saintpaulia tongwensis*	*Saintpaulia ionantha* subsp. *ionantha* 18	1985-0668 E
AF307034	*Saintpaulia* cf. *ionantha*	*Saintpaulia ionantha* subsp. *ionantha* 19	1971-0860 E
AF108748	*Saintpaulia tongwensis*	*Saintpaulia ionantha* subsp. *ionantha* 20	Pangani Falls.1 EA_NMK
AF108736	*Saintpaulia ionantha*	*Saintpaulia ionantha* subsp. *ionantha* 21	Amboni.I.2 EA_NMK
AF108732	*Saintpaulia ionantha*	*Saintpaulia ionantha* subsp. *ionantha* 22	1983-8132 K
AF108730	*Saintpaulia tongwensis*	*Saintpaulia ionantha* subsp. *ionantha* 23	Tongwe.9 EA_NMK
AF307029	*Saintpaulia diplotricha*	*Saintpaulia ionantha* subsp. *ionantha* var. *diplotricha* 24	1987-2172B E
AF324927	*Saintpaulia nitida*	*Saintpaulia ionantha* subsp. *nitida* 25	1997-0104 E
AF324928	*Saintpaulia orbicularis*	*Saintpaulia ionantha* subsp. *orbicularis* 26	1987-2177 E
AF307041	*Saintpaulia orbicularis* var. *purpurea*	*Saintpaulia ionantha* subsp. *orbicularis* 27	1958-3586B E
AF108740	*Saintpaulia orbicularis*	*Saintpaulia ionantha* subsp. *orbicularis* 28	Kwabulu.5 EA_NMK
AF108739	*Saintpaulia orbicularis*	*Saintpaulia ionantha* subsp. *orbicularis* 29	1987-1370 K
AF307043	*Saintpaulia pendula* var. *pendula*	*Saintpaulia ionantha* subsp. *pendula* 30	1998-1691 E
AF307042	*Saintpaulia pendula* var. *kizarae*	*Saintpaulia ionantha* subsp. *pendula* 31	1997-0103 E
AF307033	*Saintpaulia intermedia*	*Saintpaulia ionantha* subsp. *pendula* 32	1997-0101 E
AF307046	*Saintpaulia rupicola*	*Saintpaulia ionantha* subsp. *rupicola* 33	JS02 E
AF307044	*Saintpaulia rupicola*	*Saintpaulia ionantha* subsp. *rupicola* 34	1997-0095 E
AF108745	*Saintpaulia rupicola*	*Saintpaulia ionantha* subsp. *rupicola* 35	Simiyu 169-96-1739 NMK
AF108742	*Saintpaulia rupicola*	*Saintpaulia ionantha* subsp. *rupicola* 36	Lindquist 97002.1, EA_NMK
AF307051	*Saintpaulia velutina*	*Saintpaulia ionantha* subsp. *velutina* 37	1987-2179 E
AF307025	*Saintpaulia brevipilosa*	*Saintpaulia ionantha* subsp. *velutina* 38	1970-0909 E
AF108750	*Saintpaulia* cf. *velutina*	*Saintpaulia ionantha* cf. subsp. *velutina* 39	Dibohelo.1 EA_NMK
AF108749	*Saintpaulia velutina*	*Saintpaulia ionantha* subsp. *velutina* 40	Mgambo.4 EA_NMK
AF108733	*Saintpaulia brevipilosa*	*Saintpaulia ionantha* subsp. *velutina* 41	1995-503 K
AF108741	*Saintpaulia pusilla*	*Saintpaulia pusilla* 42	Magari.1 EA_NMK
AF307047	*Saintpaulia shumensis*	*Saintpaulia shumensis* 43	1996-2088 E
AF108746	*Saintpaulia shumensis*	*Saintpaulia shumensis* 44	Shume.1 EA_NMK
AF324930	*Saintpaulia* sp.	*Saintpaulia* sp. 45	1998-1687 E
AF324929	*Saintpaulia* sp.	*Saintpaulia* sp. 46	1993-1279 E
AF307045	*Saintpaulia* sp. ‘Kachororoni’	*Saintpaulia* sp. 47	JS01 E
AF307036	*Saintpaulia* sp. ‘Sigi Falls’	*Saintpaulia* sp. 48	1992-3183 E
AF108743	*Saintpaulia* sp. ‘Kachororoni’	*Saintpaulia* sp. 49	Lindquist 97003,C EA_NMK
AF108744	*Saintpaulia* sp. ‘Mwache’	*Saintpaulia* sp. 50	Pearce 543-94-530.1 NMK
AF307049	*Saintpaulia teitensis*	*Saintpaulia teitensis* 51	1997-0385 E
AF307048	*Saintpaulia teitensis*	*Saintpaulia teitensis* 52	C3771 E
AF108747	*Saintpaulia teitensis*	*Saintpaulia teitensis* 53	Lindquist 97001,C EA_NMK
AF108727	*Streptocarpus caulescens*	*Streptocarpus caulescens*	1971-1199 E

Name sensu Darbyshire (2006) reflects the updated taxonomy. Abbreviations are as follows: Royal Botanic Gardens, Kew - K; Royal Botanic Garden, Edinburgh - E; National Museums of Kenya, Nairobi - NMK; East African Herbarium, National Museum of Kenya, Nairobi - EA_NMK. * misidentification most likely *Saintpaulia ionantha* subsp. *ionantha* (see Discussion for further comments).

A better understanding of the biogeographic histories of Eastern Arc lineages is essential to explain how present diversity has been formed and maintained in this biodiversity hotspot over the recent geological time frame, and in the context of past climatic fluctuations. The Eastern Arc endemic plant genus *Saintpaulia* provides an ideal model to study the effects of ecosystem dynamics at high and low altitude on the diversity and distribution of tropical forest species in the Eastern Arc. A highland ancestry has been proposed for this genus [14]; however, due to the lack of a calibrated phylogeny, the timing of divergences among lineages could not be assessed. Burtt [15]–[17] recognized a total of 20 species of *Saintpaulia* as well as four varieties, but species delimitations have been questioned in two molecular studies [18], [19]. A recent revision of the genus reduced the number of species to six [20], all endemic to the Eastern Arc Mountains and coastal forests of Tanzania and Kenya. Two new species from the Uluguru Mountains, Tanzania, were recently described [21] elevating the number of species to eight. Most species of *Saintpaulia* are restricted to montane forests on a single mountain block, although *S. shumensis* B.L. Burtt occurs on two and *S. pusilla* Engl. on four mountain blocks. Only *Saintpaulia ionantha* H. Wendl. has a wider distribution over the altitudinal gradient, from coastal lowlands to montane forests. *Saintpaulia* has already attracted attention due to its high degree of endemism and commercial importance (several thousand cultivars originating from a few specimens of wild *S. ionantha* are sold under the common name of African Violet or Usambara Violet) [14], [18], [19], [22]–[25].

Here we use historical biogeographical data for *Saintpaulia* in conjunction with distributional and environmental data to further our understanding about the role of past environmental variations on current diversity patterns in the region and the possible impacts of future changes of climate and habitats. We aim to reconcile species’ phylogenetic and biogeographic histories with information on current environmental conditions to gain insights into the processes that have shaped present day distributional patterns and diversity.

## Materials and Methods

### Taxa and Gene Sampling

Our study is based on the genus *Saintpaulia* H. Wendl. (Gesneriaceae; ‘African violets’), an endemic plant of the Eastern Arc Mountains in Kenya and Tanzania. Here we adhere to the *Saintpaulia* classification proposed by Darbyshire [20] including the species from Haston et al. [21]. In particular cases where species determinations are uncertain we also refer to the original label information. We attempted to maximize the representation of *Saintpaulia* lineages using sequences from specimens of this genus present in public data repositories (e.g., GenBank). Data were available for five of the currently recognized species with the exceptions of *S. inconspicua* B.L. Burtt, a very rare species with a very limited distribution in the highlands of the Uluguru Mountains, and the two species described by Haston et al. [21]. As outgroup we used *Streptocarpus caulescens* Vatke. The genus *Streptocarpus* Lindl. (Gesneriaceae) has been hypothesized as the closest relative to *Saintpaulia* [22] and has been used to root the *Saintpaulia* phylogeny in previous studies (e.g., [14]).

There are few published molecular datasets of *Saintpaulia* and they rely on different genetic markers. Möller and Cronk [19] used ITS sequences to study the relationships and biogeography of 17 *Saintpaulia* species (of the 20 species recognized at that time) cultivated in the research collection at the Royal Botanic Garden, Edinburg. Lindqvist and Albert [14], [18] used 5S non-transcribed spacer (5S-NTS) of all but one of the species then included in the genus. More recently Caro et al. [25] sequenced chalcone synthase (*CHS*) for five out of the approximately 22 species recognized species at the time. In addition a couple of *atpB-rbcL* spacer, *trnL-F* and ITS1/ITS2 sequences were generated by Möller et al. [26] as part of a large study of didymocarpoid Gesneriaceae. Few additional data are available (e.g., [27], [28]). Among these datasets, the study of Lindqvist and Albert [14], [18] includes the largest number of *Saintpaulia* lineages. In addition, the recent taxonomic changes and the use of different voucher specimens in the various studies make it impossible to combine these datasets. For these reasons we have selected the Lindqvist and Albert [14], [18] data for our analyses, with the addition of sequences of 5S-NTS from the Möller et al. [26] that were generated using the same set of primers. Additional ITS data from Möller et al. [26] for *S. tongwensis* and *S. velutina* ware also included in the molecular clock analyses to augment overlap with the [29] dataset, used to estimate the divergence between *Saintpaulia* and *Streptocarpus* as explained below. This second matrix had larger representation of Gesneriaceae and is referred as RCZ_dataset. The [29] sequences are available in TreeBase under study number TB2: S1820 or via this link http://purl.org/phylo/treebase/phylows/study/TB2:S1820. Accession numbers for all sequences used in the present analyses that are not available through the aforementioned link are presented in Table 1.

### Phylogenetic Analysis and Molecular Dating

Calibration of a molecular clock for *Saintpaulia* is problematic due to the absence of fossils or sister lineages with disjunct distributions along well defined geological features of known age. The genus is also missing from recent analyses of Gesneriaceae that use molecular clock techniques [29]. To overcome these limitations, we have adopted an indirect calibration approach that relies entirely on already published data [29]. Due to the lack of overlap between the most taxon-rich dataset for *Saintpaulia* [14] and the datasets of [26], [29] we built two separate matrices and ran two different molecular clock analyses. The existing calibrated phylogeny containing *Saintpaulia* sister group *Streptocarpus* [29] was used to estimate the time of divergence of these two lineages. To do so, we added two taxa of *Saintpaulia*, *S. ionantha* H. Wendl. subsp. *ionantha* (as *S. tongwensis* B.L. Burtt) and *S. ionantha* subsp. *velutina* (B.L. Burtt) I. Darbysh. (as *S. velutina* B.L. Burtt), sequenced for the same gene fragments [26], to the original dataset of [29].

Two different calibration schemes (56/52 and 56/52/8) from Roalson, Skog and Zimmer [29] adding the estimated age of the clade Beslerieae+Napeantheae (ranging from 71.62 Ma to 33.65 Ma with a mean of 52 Ma in the majority of their analytical treatments) from the same study were used with the RCZ_dataset. Both calibration schemes rely on geological events (e.g., GAARLANDIA land bridge formation; see [29]) and estimates for the maximum age of the stem age of the Gesneriaceae by Bremer et al. [30]. Under the 56/52 scheme Gesneriaceae stem maximum age was constrained to 71 Ma [30] and the Gesnerieae+Gloxinieae stem lineage age was constrained between 35 Ma and 25 Ma [29]. The 56/52/8 strategy adds an additional maximum age constrain for the migration of the Gloxinieae back to South America [29]. Further details on the 56/52 and 56/52/8 calibrations are given in [29]. The data was analyzed with BEAST v1.5.4 [31] using a relaxed uncorrelated lognormal clock [32]. All calibrations based on geological events or set as maximum constraints were implemented using uniform density distributions for the *tmrca* priors; normal distribution was used for the Beslerieae+Napeantheae constraint. The resulting estimate for the age of the *mrca* of *Saintpaulia* and *Streptocarpus* was used as the calibration point in the analysis of the second dataset, which included all available *Saintpaulia* 5S-NTS sequences from [14], [18] – the LA_dataset. This calibration was applied using a normal distribution for the *trmca* prior.

Given the lack of known fossils or suitable geological calibration points for the LA_dataset, an alternative to the described dating protocol could be the use of a fixed rate of DNA evolution for the 5S-NTS with the corresponding standard deviation. However, such rate has not been established for *Saintpaulia* and close relatives. Potential use of rates estimated for other angiosperms would likely result in very incorrect age estimates as rates of molecular evolution in plants are know to be extremely variable (e.g., [33]–[36]).

Since the publication of the aforementioned datasets, taxonomy of *Saintpaulia* has been revised, so that names associated with GenBank sequences often do not match the current classification. Here we have adopted the most recent classification as proposed by Darbyshire [20]. However, we did not re-examine the voucher specimens used to generate the DNA data in the original studies; thus the old names and the specimens voucher numbers are also kept as a reference. Revising the systematics of *Saintpaulia* is beyond the scope of this study and original vouchers should be examined before formalizing any nomenclatural changes. GenBank accession numbers and relevant specimen information are shown in Table 1.

All BEAST analyses were run assuming a birth-death tree prior using the maximum likelihood starting trees built with RaxML v7.2.6 [37] on the CIPRES cluster [38]. When datasets included more than one gene, they were partitioned by gene. The GTR+Γ model of sequence evolution was used in all RaxML analyses (as recommended by the program manual); for consistency reasons the same model was used in all BEAST analyses. In the case of the LA_dataset (which included just one gene as specified above), the best fit model selected by jModeltest v0.1.1 [39] was the TVMef+Γ, hence an additional round of BEAST analyses was run using this model to make sure that use of the more general GTR+Γ did not affect the results. Analyses in BEAST were run for 10 million generations; trees were sampled every 1000 generations. Results were examined for convergence with Tracer v1.5 [40] paying special attention to ensure that effective sample size of all parameters was above 200. All datasets were realigned using the L-INS-i method in MAFFT v6 [41].

Alternative molecular clock methods available in the package r8s [42] could not be used with the LA_dataset as they require at least one fixed calibration point, and therefore r8s was not considered for our analysis. The median values of the 95% confidence intervals for the age of the *mrca* of *Saintpaulia* and *Streptocarpus* as estimated by BEAST were compared and their mean was used in the calibration of the LA_dataset assuming normal distribution.

Haplotype networks were built with TCS v1.21 [43] using the LA_dataset. The networks were assembled based on the number of mutations separating each haplotype with a parsimony probability of 95% (the default settings in TCS).

### Species Distributions Models

Distributional data for *Saintpaulia* consisted of 147 presence records covering most of the described species. Although this is the largest distributional dataset available, only two species are represented by over ten records: *S. ionantha* and *S. pusilla*. Georeferenced records were not available for the species described by Haston et al. [21]. Because all lowland populations fall within a monophyletic clade (*S. ionantha*) with no indication of hybridization with the strictly highland species (see results), we decided to merge all records of the strictly highland species of *Saintpaulia* to supply the modeling algorithm with sufficient presence records. Therefore, instead of modeling current and future climatic suitability of single species, we estimate suitability of climatic conditions for two sets of species: high elevation (strictly high elevation lineages) and low elevation (the remaining lineages).

Current and future 30Arc seconds downscaled climatic data including minimum, maximum and mean temperature and monthly precipitation were obtained from the WORLDCLIM database (http://www. worldclim.org/). Spatial downscaling is widely used to obtain data with higher spatial resolution. However, this approach assumes that the relationship between large- and small-scale climate variables is stationary over time, which is unlikely to always hold true, implying that downscaling might produce errors that could propagate across scales [44]. We are well aware of the problems with downscaled data (e.g., [45]); however, due to the lack of regional forecasting models these are the only data concerning the Eastern Arc region currently available. As there is a high level of uncertainty on how CO_2_ and other greenhouse gas emissions will evolve in the future, we used four different predictions for the potential climate changes in 2080 based on two different Atmospheric Ocean coupled General Circulation models, CGM2 and 3.1 and HadCM3, and two different emission scenarios, A1b and B2 [46]. The A1 emission scenarios family describes a future world with maximum energy requirements and specifically the A1b describes a future world with a balanced use of fossil and non-fossil sources of energy. The B2 family of emission scenarios describes a future world with lower energy requirements than A1.

In addition to the climatic data, land cover, lithology and soil data were available and included in analyses for the Tanzanian part of the *Saintpaulia* distribution. Due to the lack of future projections of these variables, we have assumed no change in 2080 when calculating future habitat suitability. However, this assumption is violated in some cases (e.g., land cover [47]) and ignoring changes may lead to positive bias in the projections. It is, nonetheless, important to include these variables as they are critical for the distribution of *Saintpaulia* and help better understand the potential effects of changes in the environment such as land use that are not strictly related to climate.

Species potential distributions were modeled using the maximum entropy method implemented in the software package MAXENT [48], [49]. This method uses presence only data and performs well when few distributional records are available [50], and it has ranked very high in a recent comparison of species distribution modeling methods [51]. To study the potential effects of future climate changes modeled distributions were inferred based on projections for 2080. Models performance was assessed by the means of ten-fold cross-validation as implemented in MAXENT. To reduce the risk of over fitting, preliminary runs were conducted with all variables. Based on these analyses, only the variables with higher contribution were selected for subsequent analyses. Final analyses were limited to the six most important variables.

In order to make sure that discussed trends in habitat availability are not biased due to algorithm choice, in addition to MAXENT we have used also BIOCLIM [52] and GAPR with best subsets [53] algorithms as implemented in the package openModeller v1.1.0 [54]. BIOCLIM and GARP analyses were applied only to the reduced set of climatic variables across the whole Eastern Arc region.

Information on all distribution modeling analyses, including model performance and relevant statistics are reported in Table 2.

## Results

### Saintpaulia Phylogeny and Biogeography

**Figure 1:**
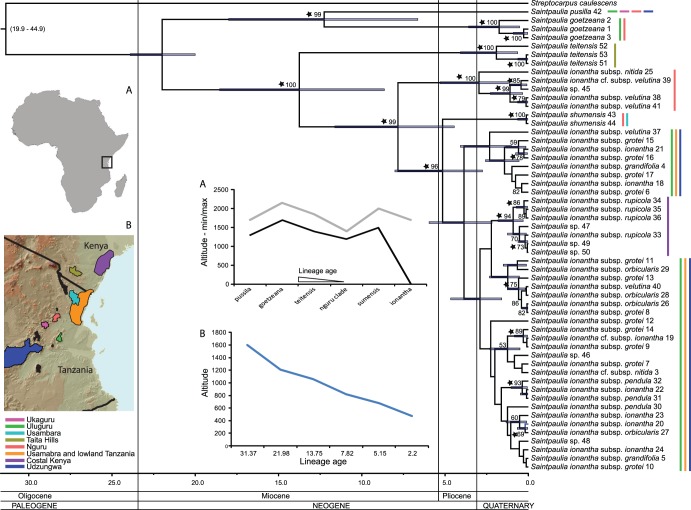
Chronogram of *Saintpaulia*. Values above branches are bootstraps from the maximum likelihood analyses; black stars show clades that receive posterior probability>95% in the BEAST analysis. Node bars show dating 95% confidence intervals (error for the *Saintpaulia – Streptocarpus* divergence given in brackets). Colors in map B and color bars in the chronogram represent geographical distributions (see legend under map B). Map A – Africa with a square showing the position of the study area. Map B – detailed map of the Eastern Arc region. Graph A – min/max altitude for each linage. Graph B – reconstructed ancestral altitude (altitude treated as continuous character) vs. lineage age. Numbers following taxa names are unique identifiers that link the specimen to the voucher and original determination listed in Table 1.

Maximum likelihood results for the LA_dataset agree with previously published phylogenies, based on maximum parsimony, of what is essentially the same dataset [14]. *Saintpaulia* monophyly was well supported and results suggested well structured lowland populations. Several specimens from the Nguru Mountains sequenced by [14], [18] originally determined as *S*. indet (voucher 1998-1687, Edinburgh), *S. brevipilosa* (voucher 1970-0909, Edinburgh), *S. brevipilosa* (voucher 1995-505, Kew), *S. nitida* (voucher 1997-0104, Edinburgh) and *S*. cf. *velutina* (voucher Dibohelo.1, East African Herbarium) formed a well supported monophyletic clade (Fig. 1). According to the current taxonomy [20], and given that original determinations are correct, these specimens belong to *S. ionantha* (subsp. *nitida* and subsp. *velutina*); thus as currently delimited, it is paraphyletic with respect to *S. shumensis*. The two *Saintpaulia* species included in the RCZ_dataset analyses also formed a well supported clade sister to *Streptocarpus*.

**Table 2:** AUC values from the different algorithms and datasets.

	BIOCLIM		GARP MAXENT AUC values*		
	Full	Full	Full	Tanzania/climate only	Tanzania
***High***					
A1B_cgcm2	0.98	0.98	0.975	0.957	0.950
A1B_hadcm3	0.99	0.98	0.975	0.957	0.950
B2a_cgcm2	0.98	0.98	0.975	0.957	0.950
B2a_hadcm3	0.99	0.99	0.975	0.957	0.950
***Low/High***					
A1B_cgcm2	0.96	0.94	0.990	0.975	0.978
A1B_hadcm3	0.95	0.92	0.990	0.975	0.978
B2a_cgcm2	0.96	0.94	0.990	0.975	0.978
B2a_hadcm3	0.96	0.94	0.990	0.975	0.978

*Average AUC values from ten-fold cross validation.

The basalmost *Saintpaulia* lineages are restricted to the high elevation montane forests as in [14]. Reanalysis in BEAST of the RCZ_dataset under the two different calibration schemes specified above resulted in very similar estimates for the age of the *mrca* of *Streptocarpus* and *Saintpaulia*: 21.92 Ma (11.17–33.67 Ma, 95% confidence interval) and 22.31 Ma (11.07–34.83 Ma, 95% confidence interval) respectively. The mean of these two estimates was used to calibrate the split of *Streptocarpus* and *Saintpaulia* in the LA_dataset assuming a normal error distribution of the age prior. The LA_dataset molecular clock analyses showed an old divergence between lowland and highland lineages. The younger lowland lineages formed several distinct populations. Most of the lowland populations diverged long before the quaternary glaciation and have persisted in the area (Fig. 1). The divergence of the Nguru *S. ionantha* specimens is significantly older than that of the major *S. ionantha* group. Analyses of the LA_dataset under the TVMef+Γ and the GTR+Γ converged to the same results (overlapping age estimates and same topology). Because the TVMef+Γ is just a specific case of the more general GTR+Γ, and the latter was used to build the starting tree for the dating analyses, only the results based on the GTR+Γ model are shown here (Fig. 1).

**Figure 2:**
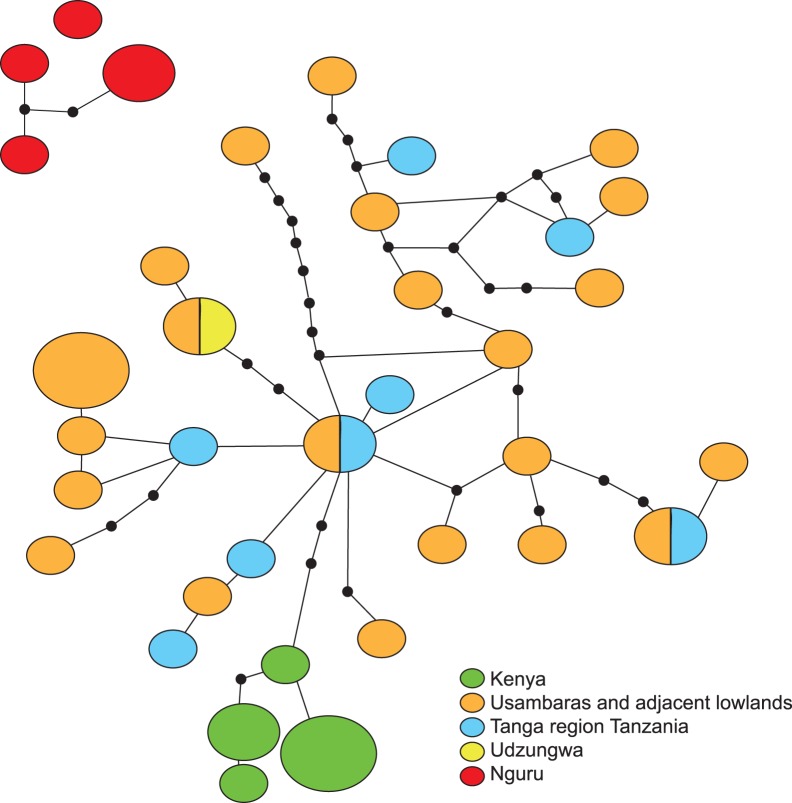
TCS haplotype network of *S. ionantha* (note that specimens of *S. ionantha* from Nguru do not form a network with the rest of the putative conspecifics; see text for further information). Size of bubbles is proportional to the number of specimens (small –1; intermediate –2; large –3). Colors represent geographical distributions.

TCS analyses also supported the separation of the Nguru *S. ionantha* populations as they did not form a network with the main *S. ionantha* clade. The Kenya coastal forest populations, *S. ionantha* subsp. *rupicola* (B.L. Burtt) I. Darbysh., are probably the result of a single dispersal into the area and there is no evidence for secondary contact with their likely source populations in the Usambara area. The populations of *S. ionantha* subsp. *ionantha* in lowland forests of Tanzania and the Usambara and Udzungwa mountains populations of *S. ionantha* from all the subspecies (subsp. *velutina*, subsp. *grotei*, subsp. *orbicularis* and subsp. *grandifolia*) found in these areas share haplotypes (Fig. 2).

### Species Distribution Models and Projected Distributions under Climatic Change Scenarios

**Figure 3:**
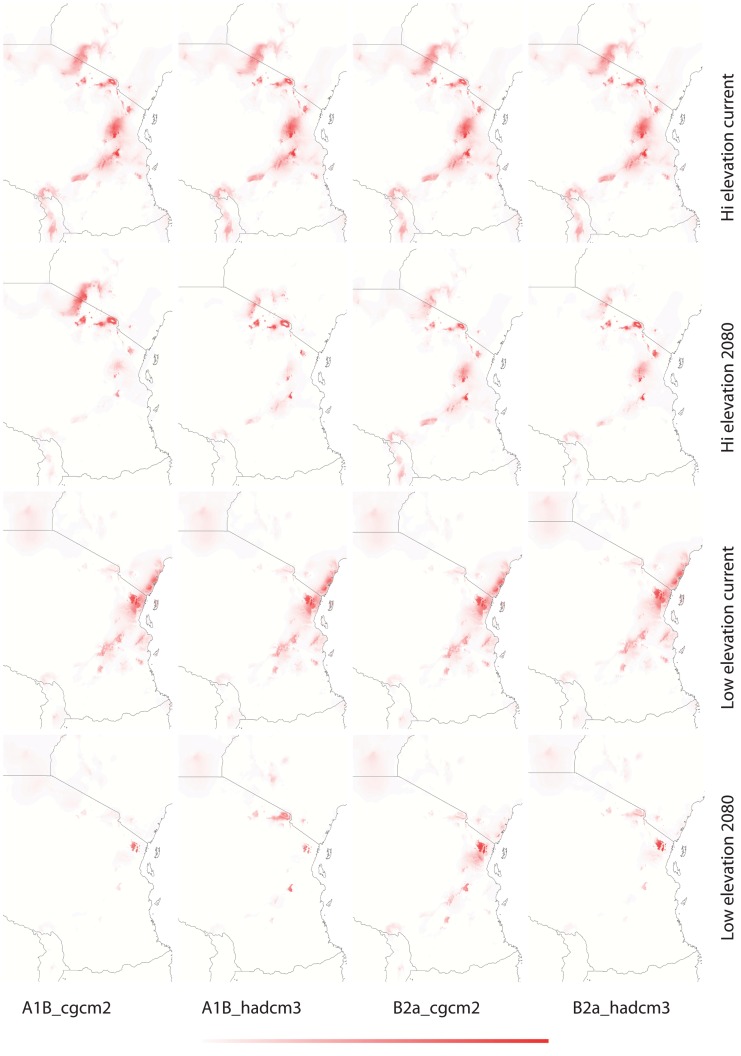
Current and future (2080) habitat suitability of *Saintpaulia* in the Eastern Arc region based on climatic data only - MAXENT. Darker color denotes higher suitability; color scale ranges from 0 (white) to 1 (red).

**Figure 4:**
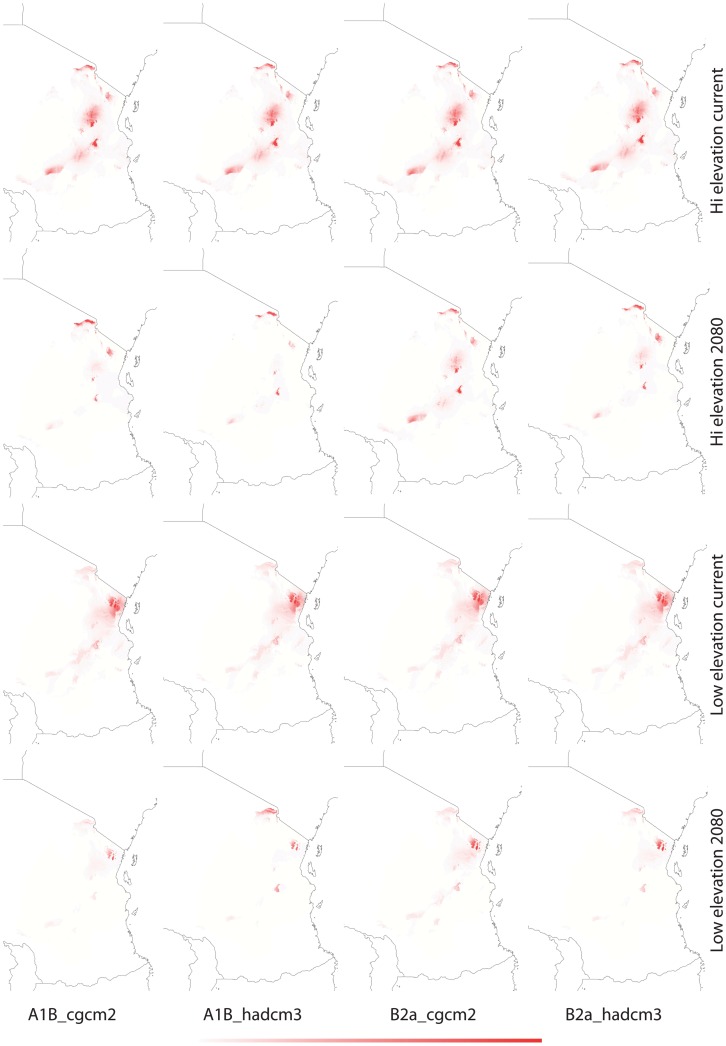
Current and future (2080) habitat suitability for *Saintpaulia* in the Tanzanian part of the Eastern Arc region based on climatic data - MAXENT. Darker color denotes higher suitability; color scale ranges from 0 (white) to 1 (red).

**Figure 5:**
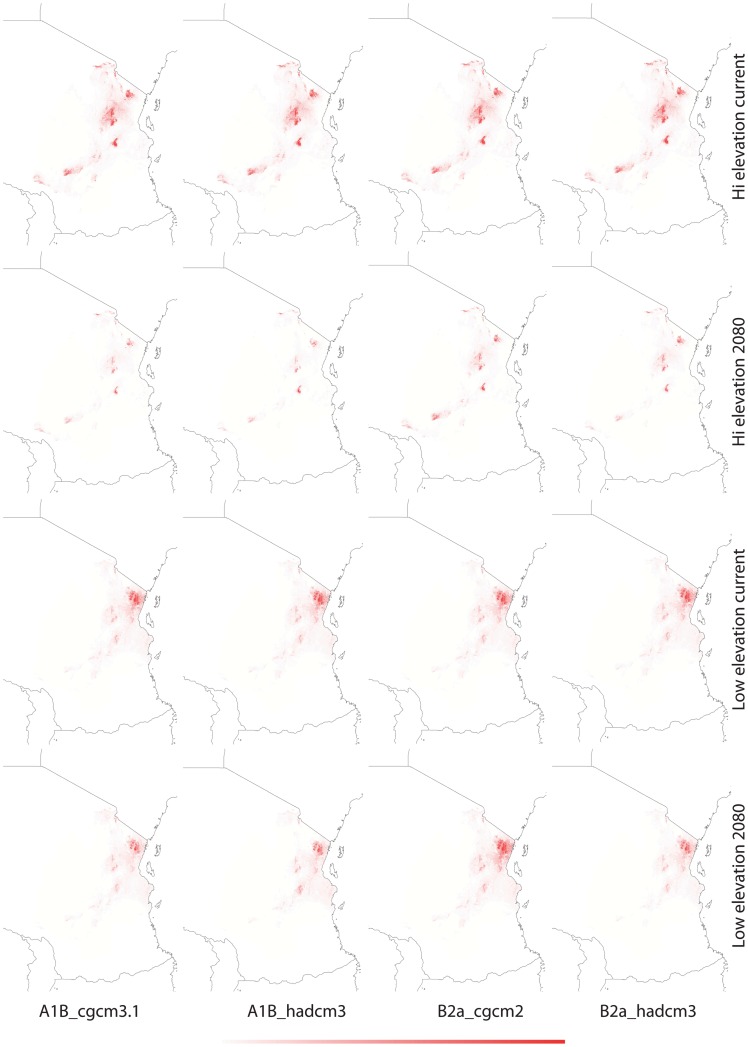
Current and future (2080) habitat suitability for *Saintpaulia* in the Tanzanian part of the Eastern Arc region with information on climate, soils, land cover and lithology added - MAXENT. Darker color denotes higher suitability; color scale ranges from 0 (white) to 1 (red).

**Figure 6:**
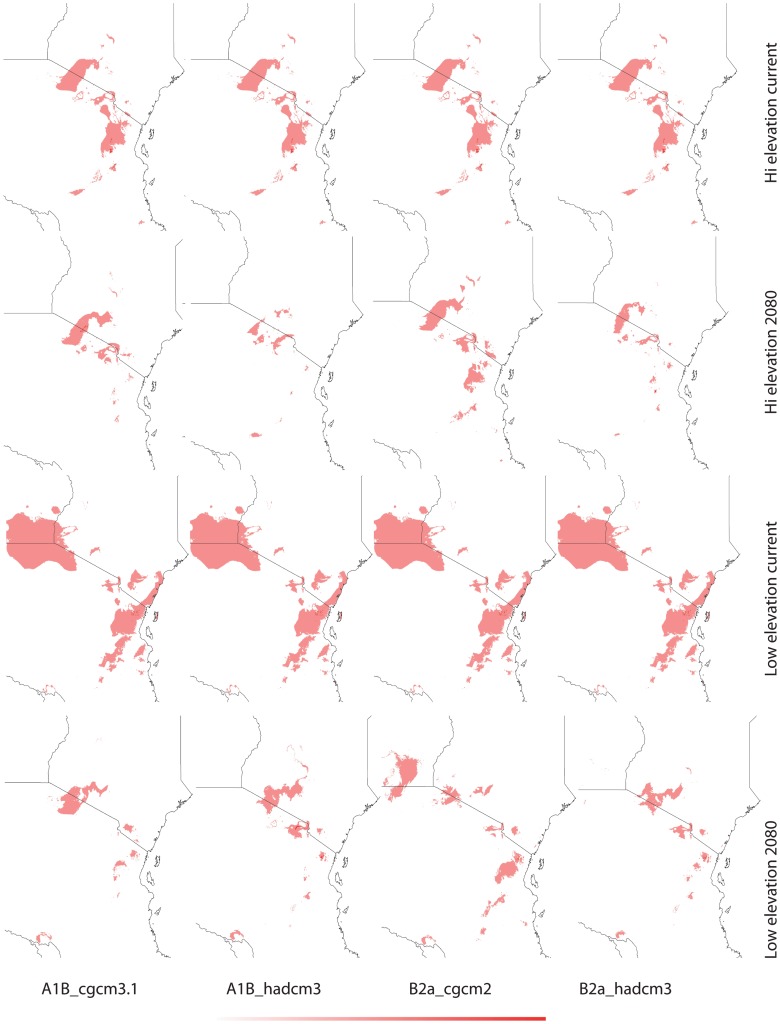
Current and future (2080) habitat suitability of *Saintpaulia* in the Eastern Arc region based on climatic data only - BIOCLIM. Darker color denotes higher suitability; color scale ranges from 0 (white) to 1 (red).

**Figure 7:**
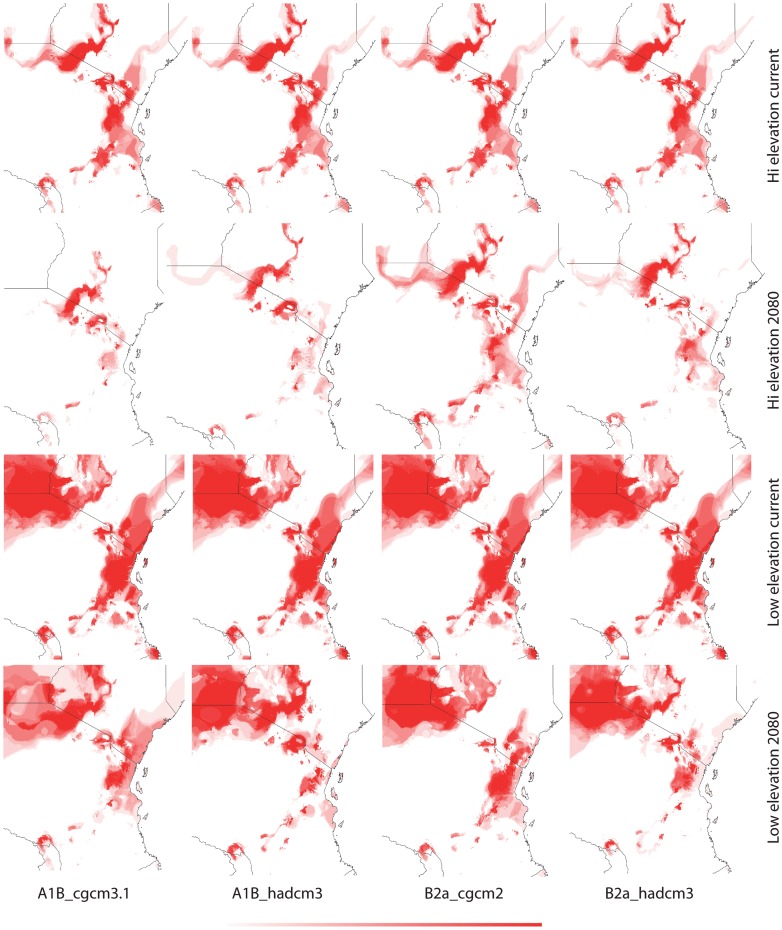
Current and future (2080) habitat suitability of *Saintpaulia* in the Eastern Arc region based on climatic data only - GARP. Darker color denotes higher suitability; color scale ranges from 0 (white) to 1 (red).

Results from tenfold cross validation in MAXENT and model performance statistics in BIOCLIM and GAPR show that models predictions are significantly different from random based on the area under the curve (AUC) of the receiver operation characteristic (ROC) (Table 2). Cross validation in MAXENT and using a subset of the distribution records as a test dataset in BIOCLIM and GARP allows us to test the internal consistency of the models; however, to evaluate their predictive power an independent evaluation dataset would be necessary. Therefore, predictions have to be treated with caution. The climatic variables with highest contribution to each model are given in Table 3. Suitable habitats under current climatic conditions differ between the high and low elevation datasets with greater spatial overlap in the Usambara Mountains (Figs. 3–5). Using different algorithms result in differences in the extent of the areas with suitable climatic conditions (Figs. 6–7), but except for differences in their size suitable areas are otherwise consistent between different methods. When the minimum and maximum altitudes of each lineage are plotted, the difference in range breadth between the high and low elevation lineages is particularly apparent (Fig. 1). Our models also identify areas with suitable climatic conditions in the southernmost parts of the Eastern Arc chain and on the younger volcanic mountains along the border of Tanzania and Kenya, Mount Kilimanjaro and Mount Meru, where *Saintpaulia* has not been found.

**Table 3:** List of the environmental variables used.

Variables selected for the analyses (with greater contribution when all variables are included)			
Full dataset and Tanzania climate only		Tanzania climate+additional variables	
*High elevation set*	*Low elevation set*	*High elevation set*	*Low elevation set*
Precipitation January	Precipitation April	Landcover	Landcover
Precipitation July	Precipitation July	Lithology	Lithology
Precipitation August	Precipitation September	Organic carbon	Organic carbon
Precipitation September	T max July	Precipitation July	Precipitation July
T max July	T min January	T min July	Precipitation September
T min September	T min December	T min November	Precipitation October

Precipitation layers contain the average monthly precipitation in mm; T max - average monthly maximum temperature in °C multiplied by 10; T min average monthly minimum temperature in °C multiplied by 10.

Suitable habitats for both groups of species under all climatic scenarios are expected to be significantly reduced by 2080 (Figs. 3–5). Practically all suitable habitats at lower elevation are expected to disappear while areas with suitable climatic conditions on the younger volcanic mountains along the border of Tanzania and Kenya where the plant is not present (Mount Kilimanjaro and Mount Meru) will increase. These results are congruent with recent findings suggesting that impacts of climate change in mountain biodiversity can be more significant in lowlands than in highlands [55], [56]. Using alternative algorithms to model species distributions and habitat suitability resulted in predictions showing the same general trends under the different climate change scenarios that we considered (Figs. 3–7).

The predicted distribution of *Saintpaulia* under both current and future conditions changed when additional information on soils, land cover and lithology was added (Fig. 5). The size of the available habitats and the associated probabilities of species occurrence diminished. Although non-climatic information was available only for the Eastern Arc region of Tanzania, we expect to see the same pattern for the Kenyan populations.

## Discussion

Our results support the hypothesis of montane origins of *Saintpaulia* [14] and place its origin in the Oligocene (Fig. 1). This coincides with the initial processes of fragmentation of the tropical forests [57] that covered most of the African continent up to about 32 Ma ago [10]. The Eastern Arc mountains were already present at this time (the basal structures of the mountains are at least 30, and perhaps more than 100 million years old, but final tilting resulting in the highest elevations may be as young as 7 Ma [58]) and many of their forest species descend from lineages inhabiting ancient tropical forests [10]. The early divergence of *Saintpaulia* and *Streptocarpus* is probably a signature of these early fragmentation processes. Wetter conditions during the Miocene and the corresponding increase in forest extent have allowed the spread of plant species through Eastern Africa and have fostered potential species exchange between East and West African tropical forests (e.g., [10], [59], [60]).

The Eastern Arc origins of *Saintpaulia*, its limited dispersal abilities, together with its tight association with stony stream banks in montane forests (naturally a very patchy habitat with limited extent), have posed additional limitations on species ranges. The majority of *Saintpaulia* species are restricted to a narrow altitudinal band (Fig. 1) of the montane forest throughout the Eastern Arc. This distributional pattern combined with phylogenetic results that reveal a highland ancestry suggest that niche conservatism (the tendency of closely related species to retain ecological traits of their common ancestor, resulting in similarity of their niches [61]) may be the mechanism that has maintained most of the *Saintpaulia* species’ distributions over time. Phylogenetic niche conservatism and its effects on species distributions and other important aspects related to species biology have been widely discussed in recent literature (e.g., [62]–[65]). Therefore, the warmer and wetter conditions during the Miocene climatic optimum 17–15 Ma ago [57] may have not resulted in expansion of *Saintpaulia* distribution beyond the Eastern Arc region. Alternatively, if such expansion took place and *Saintpaulia* species/populations were present outside the Eastern Arc they were driven to extinction toward the end of the Miocene/beginning of the Pliocene when the climate in Africa became drier with the formation of the Antarctic ice sheet [66]. At the same time grasslands began increasing in prevalence around the region [59], [67], thus preventing later recolonization by *Saintpaulia* species. These climatic oscillations coincided with divergences of the older lineages of *Saintpaulia* and the appearance of species endemic to the northern mountains of the Arc (Fig. 1). Further forest fragmentation starting 8–5.4 Ma ago [10] may be the reason for the split of Nguru populations of *S. ionantha* subsp. *nitida* (voucher 1997-0104, Edinburgh) and *S. ionantha* subsp. *velutina* (voucher 1970-0909, Edinburgh and 1995-505, Kew).

The aim of the present phylogenetic analyses is to provide a time frame for the diversification of *Saintpaulia* lineages. The limited data availability does not allow studying relationships in great detail and we do not intend to address taxonomic questions; therefore possible implications for systematics are not central for the discussion. It is, however, important to mention that a systematic revision in a phylogenetic context may be necessary as our results suggest that the Nguru populations of *S. ionantha* subsp. *nitida* and *S. ionantha* subsp. *velutina* (as *S. brevipilosa* in [14], [18]) may be a different species than *S. ionantha*. In the case of *S. ionantha* subsp. *nitida,* distributional patterns are consistent with this hypothesis. Phylogenetic data suggests that *S. ionantha* subsp. *nitida* is endemic to the Nguru mountains, whereas the *S. ionantha* cf. subsp. *nitida* specimen from the Tanga area (voucher Kwamtili.4, East African Herbarium) is likely a misidentification [18] and most probably belongs to *S*. *ionantha* subsp. *ionantha* (R. Gereau in litt. 2012). Darbyshire acknowledged inconsistencies between the classification that he was proposing and molecular phylogenies [20], but he argued that morphological similarities were large enough to establish the changes. At the very least, to address these inconsistencies a re-examination of the voucher specimens from Nguru and sequencing of additional populations will be required in the future.

Towards the end of the Pliocene *S. ionantha* increased its range and successfully expanded into lowland areas covered with tropical forests. The wider distribution of *S. ionantha* and particularly its wide altitudinal range indicate greater ecological plasticity. This is likely a result of adaptation to different climatic conditions leading to niche evolution in this species. Results from species distribution modeling, although suffering from many limitations, also support this conclusion. There is a tendency of overlap for the distribution of potential habitats of *S. ionantha* and the strictly highland species, but, the younger *S. ionantha* also encompasses the adjacent geographical and climatic space unsuitable to its highland congeners (Fig. 1, 3–5).

By the middle of the mid-Miocene lineages endemic to different mountain massifs were already established, and in the Pliocene lowland areas were colonized. Deep divergences among the lowland *S. ionantha* populations provide evidence for long term presence in the lowland, contradicting the traditional Pleistocene refugia model as these lineages have persisted in the area throughout the Pleistocene glaciation cycles. Thus, the montane refugia model may be relevant only to pre-Pleistocene events, as suggested by Fjeldså and Bowie [12].

*Saintpaulia* taxa are tropical forest understory plants and are never found outside forest in nature, with the exception of *S. ionantha* subsp. *rupicola*, which frequently grows in rather exposed habitats in coastal Kenya. Therefore, its presence throughout the Pleistocene in coastal and other lowland areas of Kenya and Tanzania provides strong evidence for the continuous presence of forests in the region. These forests were probably highly fragmented and separated by savannahs during glacial maxima but large enough to allow the survival of many forest species in an area that was otherwise unstable. During wetter periods when forests expanded, some of these fragmented populations likely underwent expansions and this has presented opportunities for secondary contact and hybridization. This may explain the observed pattern of shared haplotypes between lowland populations from different subspecies of *S. ionantha,* which otherwise show old divergences (Fig. 2). Periods of populations’ expansion and contraction (with extinction of some populations and their haplotypes) also fit the observed reticulatations in the haplotype network and the large number of reconstructed missing haplotypes (Fig. 2). This process of lineage differentiation is similar to the one described from leaf chameleons [13] and also takes place on the periphery of the stable areas as hypothesized by the stability theory.

The origin, maintenance and distribution of African violets in Eastern Arc Mountains and Coastal Forests seems therefore connected to past climatic stability in the highlands and changes in the lowlands. Thus, how will climate change affect African violets during this century? All *Saintpaulia* species are at high risk due to habitat degradation and environmental changes [23], [24]. Although our future projections address only the climatic component of environmental change, habitat degradation due to human activities is very severe in this region [47], [68], and as both Kenya and Tanzania face population growth and associated development it is unlikely that this tendency will change soon. The importance of non-climatic variables was reflected in the niche modeling results for Tanzania where some data were available (Fig. 5); as a result of landuse changes some of the climatically suitable areas are no longer habitable for *Saintpaulia*. Most of the conservation effort in the region has been traditionally focused on the mountain areas, although there is a recent surge in establishment of protected areas in the coastal and other lowland forest [69]. Evidence for lowland micro-refugia indicates that lowland populations may have higher chance to survive future climate changes if putative micro-refugia are considered when designing protected areas. However, most of the lowland areas with suitable climatic conditions for *Saintpaulia* are already much altered (transformed for agricultural use). Even worse, projections of climatic suitability show rapid collapse of the suitable habitats in a dramatic loss of genetic diversity and virtual extinction of some populations within the next 70 years (e.g., all Kenya haplotypes may go extinct). This is likely due to the fact that the expected climate change for 2080 may bring levels of green house gases and global temperatures that do not have analogs during the Pleistocene interglacial periods [70]. Significant warming is expected for the East African region and predictions also show increased net rainfall and stronger seasonality in this region [71]. As a result some of the lowland micro-refugia may experience environmental changes which would make these areas unsuitable for *Saintpaulia*. Under this scenario additional measures may be needed to ensure survival of lowland lineages; establishment of protected areas alone will not be able to solve the problem.

In contrast to the coastal forests, the montane areas seem to be in a much better position. Several of the high elevation areas with suitable climates are already protected and, more importantly, according to our results, expected changes in climate will not cause shifts of suitable conditions beyond existing reserves. However, the surface area of mountain regions with suitable conditions will decrease significantly, resulting in increased vulnerability of *Saintpaulia* species and likely other plants and animals adapted to the same environmental conditions.

Our results on future impacts of climate change in African violets should be interpreted with caution. As in other tropical mountains, where local climates are decoupled from the global weather trends due to regional phenomena, climatic data available for reconstructing current and future climatic trends is scarce [72]. Moreover, predicting biodiversity responses to climate change in the tropics, where data on species occurrence are scarce, is challenging enough [73] and the accumulation of climatic data is only one of many challenges affecting the accuracy of niche modeling predictions in these regions. However, our results provide a useful first approximation to a better understanding of the processes that have shaped diversity in the Eastern Arc and also show general tendencies for the future changes that may be a helpful guide for better conservation planning.

In summary, we present evidence that pre-Quaternary speciation processes and stable environmental conditions have been key factors for the high levels of biodiversity in the Eastern Arc Mountains, as proposed by the long-term stability scenario. However, the role of the upper parts of these mountains as climatic refugia for lowland lineages, by preventing extinctions during the Pleistocene, may have also contributed to augment and maintain the extraordinary species diversity of this area. We also find evidence for the existence of lowland micro-refugia during the Pleistocene, but further investigation is necessary to characterize them better. Such micro-refugia have likely been a paramount for the survival of the lowland *Saintpaulia* populations during the Pleistocene climatic fluctuations, and should be given priority for conservation. Our analyses suggest that there is not any single factor, but a combination of biogeographical factors, that explain why tropical mountains are currently areas of high biodiversity concentration.
